# Abdominal ultrasonography in HIV/AIDS patients in southwestern Nigeria

**DOI:** 10.1186/1471-2342-8-5

**Published:** 2008-02-29

**Authors:** Millicent O Obajimi, Mojisola O Atalabi, Godwin I Ogbole, Adenike T Adeniji-Sofoluwe, Atinuke M Agunloye, Ademola J Adekanmi, Yvonne U Osuagwu, Sefiat A Olarinoye, Mojisola A Olusola-Bello, Ayotunde O Ogunseyinde, Yetunde A Aken'Ova, Isaac F Adewole

**Affiliations:** 1 Department of Radiology, University College Hospital, Ibadan, Nigeria; 2 Department of Haematology, University College Hospital, Ibadan, Nigeria; 3 Department of Obstetrics and Gynaecology, University College Hospital, Ibadan, Nigeria

## Abstract

**Background:**

Though the major target of the HIV-virus is the immune system, the frequency of abdominal disorders in HIV/AIDS patients has been reported to be second only to pulmonary disease. These abdominal manifestations may be on the increase as the use of antiretroviral therapy has increased life expectancy and improved quality of life. Ultrasonography is an easy to perform, non invasive, inexpensive and safe imaging technique that is invaluable in Africa where AIDS is most prevalent and where sophisticated diagnostic tools are not readily available. Purpose: To describe the findings and evaluate the clinical utility of abdominal ultrasonography in HIV/AIDS patients in Ibadan, Nigeria

**Methods:**

A Prospective evaluation of the abdominal ultrasonography of 391 HIV-positive patients as well as 391 age and sex-matched HIV-negative patients were carried out at the University College Hospital, Ibadan.

**Results:**

Of the 391 cases studied, 260 (66.5%) were females; the mean age was 38.02 years, (range 15–66 years). The disease was most prevalent in the 4th decade with an incidence of 40.4%. Compared with the HIV-negative individuals, the HIV+ group of patients had a significantly higher proportion of splenomegaly (13.5% vs. 7.7%; p < 0.01), lymphadenopathy (2.0% vs. 1.3%; p < 0.70), and renal abnormalities (8.4% vs. 3.8%; p < 0.02). There were no differences in hepatic and pancreatic abnormalities between the HIV+ and HIV- groups. There were significantly fewer gallstones in the HIV+ group (1.4% vs. 5.1%; p < 0.01).

**Conclusion:**

AIDS is a multi-systemic disease and its demographic and clinical pattern remains the same globally. Ultrasonography is optimally suited for its clinical management especially in Africa. Its accuracy and sensitivity may be much improved with clinico-pathologic correlation which may not be readily available in developing countries; further studies may provide this much needed diagnostic algorithms.

## Background

The Acquired Immune-Deficiency Syndrome (AIDS), the clinical entity resulting from HIV infection is an increasingly important disease that has become a social phenomenon. The Sub-Saharan African region is by far the worst affected in the world by the AIDS epidemic. It is actually home to over 60% of all people living with HIV [1]. Though the major target of this deadly virus is the immune system, the frequency of abdominal disorders in patients with AIDS has been reported to be second only to pulmonary disease [2-4]. These abdominal manifestations may now be on the increase as the discovery and use of antiretroviral therapy has prolonged life expectancy and improved quality of life [2,5].

HIV/AIDS is known to have a wide variety of clinical manifestations from involvement of various organs. Ultrasonography (US) is a versatile imaging tool, which can evaluate most of the abdominal organs affected by the disease; furthermore, it can guide biopsies allowing the cytohistological and microbiological investigations needed to obtain a definitive diagnosis.

Other imaging methods particularly Computed Tomography (CT) can explore these organs in more detail than US. However, CT may often be considered a second choice in abdominal imaging for the following reasons; it utilizes radiation, it is more expensive, less readily available and often yields results comparable to US [3-5].

This is particularly true in developing countries where the absence or the high cost of the procedure makes abdominal US a suitable alternative diagnostic tool in the radiological investigation of HIV infected individuals.

To our knowledge, there is a dearth of reports in the West African Sub-region on imaging in HIV/AIDS especially in sonographic evaluation of the abdomen in this large population of HIV/AIDS. The purpose of this prospective study is to describe the abdominal findings in these patients and evaluate the clinical utility of abdominal US in HIV/AIDS patients in Ibadan, a southwestern town of Nigeria.

## Methods

Abdominal ultrasound scans were prospectively performed over a one-year period (April 2005–March 2006) on 391 consecutive eligible HIV-positive adults (aged 15–66 years) referred from the Antiretroviral Clinic at the University College Hospital, Ibadan to the Department of Radiology for routine diagnostic imaging. At our facility, HIV-positive patients routinely have a chest X-ray and an abdominal US for early detection of abnormality and achievement of a base line. During the same period, 391 abdominal ultrasound examinations were performed on HIV-negative adults who were enrolled as age and sex-matched controls. These included relations of patients, hospital staff and patients admitted for non-abdominal illnesses.

The ultrasound scans were performed after an overnight fast of at least 12 hours with patients lying supine and using a 3.5–5.0 MHz frequency curvilinear probe on an ALOKA 1700-SSD ultrasound machine. Non-fasting patients, children, and patients with incomplete ultrasound examinations were excluded from the study. Two radiologists performed all sonographic examinations.

The presence of the following abnormalities were noted, splenomegaly; (with or without hypo or hyperechoic lesions), hepatomegaly; (with or without single or multiple focal lesions), lymphadenopathy, gallbladder and bile duct abnormality, ascites, renal abnormalities with diffusely increased echogenicity, pancreatic changes, and biliary duct dilatation. The extrahepatic bile duct was identified at the level of the portal vein, where the hepatic artery crosses perpendicularly between them. When bowel gas obscured a part of the suprapancreatic segment, the patient was asked to take several deep breaths and hold the inspiratory phase. Color Doppler sonography was used to confirm the identification of the vascular and ductal anatomy. The common bile duct was measured in the most distal aspect of the head of the pancreas. In this location, anteroposterior measurements from inner border to inner border were obtained.

The radiologist initially recorded the data on paper and later transferred it to a computer, where it was stored throughout the period of the study before statistical analysis.

The following definitions were used, lymphadenopathy: nodes larger than 1 cm in diameter [6], splenomegaly: spleen larger than 12 cm at its longest axis [7], hepatomegaly: liver measuring more than 15 cm at its longitudinal axis, extrahepatic duct dilatation: common bile duct diameter (CBD) >7 mm. Electronic calipers were used for all measurements on the ultrasound machine.

Demographic data were obtained from patients' record file. Each patient gave an informed consent.

### Data Management

SPSS 11.0 for windows software (SPSS, Inc. Chicago, Illinois) was used for data analysis. Continuous variables were expressed as mean ± standard deviation with student T-test analysis for comparison. Categorical variables were expressed as percentages and comparison was by chi-square analysis. Two tailed p-value < 0.05 was considered significant.

## Results

Of the 391 cases, 260 (66.5%) were females; the mean age was 38.02 years, (range 15–66 years). The modal age group was the 4th decade with a frequency of 40.4% (Table 1). Of the control group, 64% were females and 36% males with a mean age of 36 years (age range = 15–64 years).

**Table 1:** Age group distribution in HIV+ Patients

**Age-group(years)**	**Frequency**	**Percentage (%)**
10–19	3	0.8
20–29	78	19.9
30–39	158	40.4
40–49	89	22.8
50–59	52	13.3
≥60	11	2.8
Total	391	100.0

### Sonographic Findings

The significant abdominal US findings seen are shown in Table 2. Of the study population normal abdominal finding were comparable in both groups. Splenomegaly was common in both groups, although it was considerably more common in the HIV+ group; 57(14.6%) compared with 30(7.7%) in the HIV- group (p < 0.01). The spleen showed homogeneous enlargement except in two of the HIV+ cases, which showed solitary hypo-echoic areas.

**Table 2:** Abdominal Ultrasound Findings in 782 Nigerian Adults*

HIV Status Number	HIV + 391		HIV-(Control) 391		P-value
**Ultrasound Finding**	**Freq**	**%**	**Freq**	**%**	
Normal	228	58.3	256	65.5	0.045
Splenomegaly	57	14.6	30	7.7	0.011
Hepatomegaly	52	13.3	58	14.8	0.381
Renal parenchymal disease	33	8.4	15	3.8	0.025
Extrahepatic duct dilatation	10	2.6	0	0	-
Lymphadenopathy	8	2.0	5	1.3	0.701
Cholelithiasis	6	1.5	20	5.1	0.006
Hydronephrosis	5	1.3	5	1.3	0.844
Ascites	5	1.3	22	5.6	0.001

* Since some patients had more than one finding the total frequency is greater than the number of patients.

**Figure 1:**
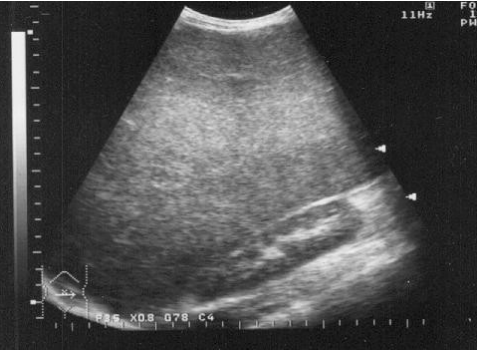
Longitudinal sonogram of liver showing hepatomegaly and increased parenchymal echogenicity with posterior shadowing (*) compatible with fatty infiltration.

Enlargement of the liver was also not significantly different in both groups: 52(13.3%) in cases versus 58(14.8%) in controls, these patients showed mostly non specific findings such as high parenchyma echogenicity compatible with fatty infiltration of the liver (Fig. 1). Evidence of cholelithiasis was noted in 6(1.5%) HIV+ cases but was significantly more in the control group, being present in 20 of 391(5.1%) patients (*p *< 0.01). Extrahepatic bile duct dilatation was also noted in 10 of 391 (2.6%) cases with a mean of 4.28 ± 1.18 mm. The width of the common bile duct ranged from 1.0 to 8.6 mm among HIV+ cases and 1.0 to 6.5 mm among the control group. No extrahepatic bile duct dilatation was recorded in the control group.

**Figure 2:**
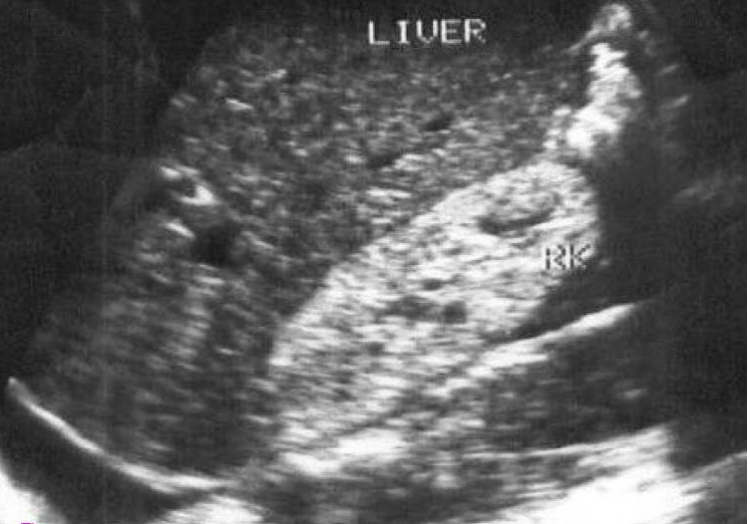
**HIV nephropathy in a 34-year-old man. **Longitudinal sonogram demonstrates normal sized right kidney with increased cortical echogenicity. Diffuse increased cortical echogenicity is associated with a poor prognosis.

Renal US findings were diverse, renal parenchymal changes as evidenced by increased echo texture of both cortex and medulla (Fig. 2) were recorded in normal sized kidneys in 33(8.4%) of HIV+ cases compared with 15(3.8%) in controls which showed similar changes (*p *< 0.03). Biochemical tests confirmed increased Urea level in 27% (9\33) of the HIV+ patients while Creatinine level was equally high in 4 of the 33 patients corresponding to those with the highest urea levels. Other renal findings in both groups include hydronephrosis, nephrolithiasis, and simple renal cysts. However the difference was not statistically significant. There was also no significant difference in the renal length in both groups (Table 3). One patient in the HIV+ group had a congenital absence of the right kidney while crossed ectopia was noted in another.

**Table 3:** Renal measurements of 782 patients

**Renal Dimensions**	**Right kidney**			**Left kidney**		
	HIV+	HIV-		HIV+	HIV-	
	
	**Mean ± SD**	**Mean ± SD**	p-value	**Mean ± SD**	**Mean ± SD**	p-value
	
Length	10.55 ± 0.97	10.60 ± 1.18	0.517	10.73 ± 1.11	10.64 ± 0.87	0.207
AP diameter	4.32 ± 0.68	4.53 ± 1.07	0.001	3.97 ± 0.31	4.07 ± 0.53	0.001

**Figure 3:**
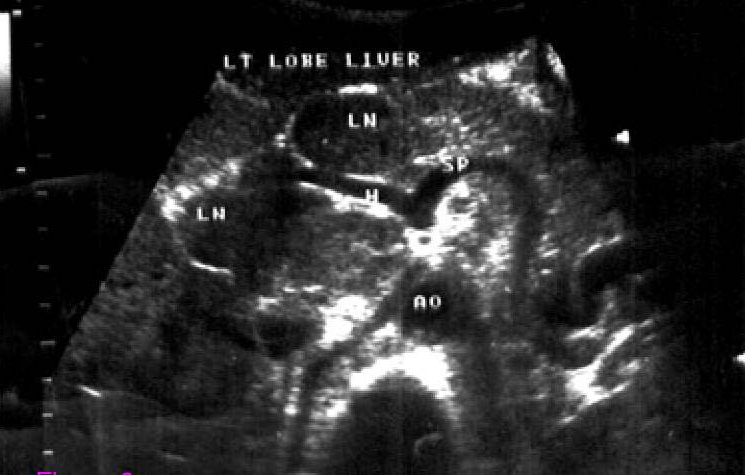
**Transverse sonogram showing extensive periportal adenopathy in a HIV+ patient with non-Hodgkin lymphoma.** AO-aorta, H-hepatic artery, SP-splenic artery, LN-lymph node.

**Figure 4:**
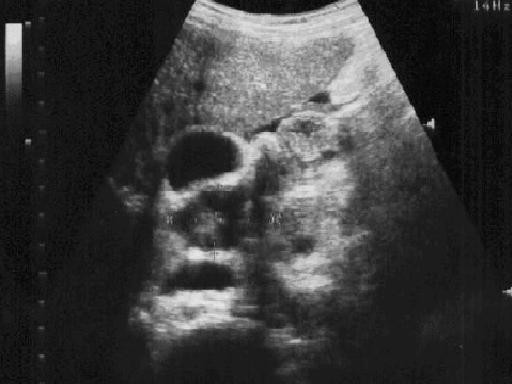
Longitudinal sonogram of liver showing a large retroperitoneal lymph node adjacent to the gall bladder in a 27 year-old asymptomatic HIV+ patient.

Enlarged para-aortic and periportal lymph nodes were more common in the HIV+ group having eight (2.0%) versus five (1.3%) in the HIV-seronegative group (Figs. 3 and 4). Free fluid (ascites) within the abdomen was seen more frequently in HIV-seronegative patients; 22 of 391(5.6%) against five of 391(1.3%) in HIV+ patients.

## Discussion

HIV/AIDS has now reached epidemic proportion in all Sub-Saharan African countries endangering not just the lives of its victims but also the social and economic fabric of society. Most health institutions in the sub region including Nigeria are not adequately equipped to properly evaluate the HIV/AIDS patient and the complications that often arise from the condition. Its infectious and non-infectious complications can be overwhelming and may be impossible to diagnose accurately in developing countries because of lack of diagnostic facilities [3-5]. Evaluation of the abdomen by ultrasound is the alternative tool to CT scan, producing cross-sectional images of high diagnostic quality. Although US does not provide a definitive diagnosis, it may show areas of abnormal anatomy and pathology that may facilitate achieving a tissue diagnosis or add further support to the decisions on commencing empirical treatment. This is highlighted by the spectrum of findings in our study as listed in Table 3. The female prevalence and mean age of 38.02 years shows a slight variance from a comparable study by Tshibwabwa et al in a similar environment [3]. Their study showed a higher male prevalence and a lower mean age. Findings in our study confirm previous documentation of the wide range of abdominal manifestations of HIV/AIDS and reveal that while abdominal abnormalities occur frequently, they are usually nonspecific, with splenomegaly, lymphadenopathy, biliary tract abnormalities, and hepatomegaly occurring most commonly. Splenomegaly was the most common abnormal finding in our study, with a frequency of 13%. This relatively high incidence agrees with previous reports [3,5,8,9]. Yee et al [10] and Geoffray et al [11], identified splenomegaly in 45% and 32.5% of their patients respectively. As is the case with the non-AIDS population, hepatosplenomegaly is a nonspecific finding. Other abnormalities of the spleen in our study occurred far less commonly. The two cases of focal hypoechoic splenic areas may have been due to splenic lymphoma. Focal splenic lymphomas are commonly depicted as a hypoechoic lesion and are often seen in association with splenomegaly [12] as in the cases identified.

However, the echogenicity of the remaining cases of splenomegaly were essentially homogeneous. In the category of patients with hepatomegaly, 49 of 52 (94%) were homogeneous. The only solid hyperechoic mass recorded was not biopsied for lack of appropriate facility. This finding is at variance with the study from central Africa, [3] where intrinsic mass lesions, namely AIDS – related lymphoma, Kaposi sarcoma of the liver, diffuse nodular regenerative hyperplasia, multiple hyperechoic nodules from extra pulmonary pneumocystitis carinii, and mycetic abscesses were found. The absence of these hepatic changes in our patients may suggest an improved quality of life consequent to the administration of the Highly-Active Antiretroviral Therapy (HAART). There was however sonographic evidence of increased parenchymal density in 35% of this group of patients. The latter is compatible with the well documented fatty changes in AIDS patients [3,5,8,9]. According to Fortgang et al, [13] this fatty change of the liver has a low sonographic diagnostic accuracy. This could account for the possibility of underreporting in our study.

Gall bladder wall thickening was not associated with the presence of calculi. Acalculous cholecystitis was also not recorded. Extrahepatic dilatation was found in 10(2.6%) patients; this finding has been reported as a sequel of AIDS Related Sclerosing Cholangitis (ARSC) [4]. However, such patients had associated findings of gall bladder wall thickening which were not demonstrated in our cases. Since these patients had no symptoms referable to the biliary system, no further diagnostic procedures were performed. The cause of this finding therefore remains unknown.

Gall stones were seen significantly less in the HIV+ group, similar findings were reported by Tshibwabwa et al [3] in central Africa consequently this pattern is probably common to all sub-Saharan African countries.

Ultrasound is valuable in the assessment of lymph nodes, with a 3.5 MHz. transducer; deep lymph nodes can be evaluated. Ultrasound allows assessment of location, number and sizes of pathological lymph nodes. It also permits evaluation of their shape, presence or absence of hilum/mediastinum. The lymph nodes recorded in our study were greater than 1 cm, mostly oval shaped with an echogenic hilum and a narrow symmetric cortex suggesting that they were benign. An ultrasound guided fine needle aspiration could have further characterized these nodes, but this could not be carried out in our centre because of unavailability of appropriate needles.

Ascites was reported more frequently in the HIV seronegative group. Perhaps the higher frequency in this group is in part due to selection bias, in that the control group was largely made up of patients on admission in the hospital. These were patients with surgical or other medical conditions who required screening for HIV as part of their diagnostic laboratory work up. Healthy individuals in our environment, usually even with counseling do not readily submit themselves for HIV screening. The higher incidence of ascites in the non-HIV population is therefore most likely due to other pathological causes such as malignancy and cirrhosis, which are the commonest known risk factors.

The association of renal abnormality with HIV has been known since the early days of the disease [13]. In 1984, Rao et al [14] described progressive nephropathy in adults with HIV/AIDS, characterized by proteinuria and renal failure. HIV-associated nephropathy may develop in patients with asymptomatic HIV infection, AIDS-related complex, or AIDS. Patients typically have mild hypertension and large kidneys and then early and rapidly progressive renal failure. Studies now show increased prevalence of renal complication because of prolonged survival and increased frequency of HIV transmission among Intravenous drug abusers [15-18]. The sonographic correlates of this disease are nephromegaly and increased cortical echogenicity [3,5,14,18]. The number of HIV+ cases in our study who showed such renal abnormality was more than double the number of the controls. However, overall renal sizes were within normal limits among both groups (Table 3). Renal failure was evident in a quarter of the cases with parenchymal changes. Nonetheless, a direct correlation between the extent of renal disease and the degree of echogenicity does not exist [19]. This strongly contrasts with the finding of previous studies [10,12,19] which found nephromegaly to be associated with HIV nephropathy, even though this finding is most common among black males [20,21]. The higher female prevalence in the study group may have accounted for this.

Our data supports the fact that manifestations and changes seen on abdominal ultrasound of HIV/AIDS patients appear to be uniform across countries in sub-Saharan Africa. However a wide range of features are seen in both HIV+ and HIV- individuals which in the absence of histological confirmation makes the clinical usefulness of ultrasound imaging quite limited. In experienced hands the radiologist may be able to provide a narrow range of diagnostic possibilities that would enhance patient management and care in African communities where the burden of HIV/AIDS remains astonishingly high.

## Conclusion

Ultrasonography is a versatile tool for evaluating abdominal organs affected by HIV/AIDS. The sonographic findings in HIV/AIDS patients in Ibadan, Nigeria are comparable to that from other sub-Saharan African communities. However, provision and availability of sufficient clinico-pathologic data in the future would improve the quality of ultrasonographic diagnosis and treatment in these patients.

## Competing interests

The author(s) declare that they have no competing interests.

## Authors' contributions

OMO and AYA conceived the study. AOM and AAM participated in its design.  OYU and AAJ performed the ultrasound examinations. OSA and OMA participated in the recruitment of subjects and collated the data. AYA and IFA coordinated the recruitment of subjects. OMO and AOA organized the ultrasound examinations. OMO, OGI and AAT managed the data, performed statistical analysis and drafted the manuscript. All authors read and approved the final manuscript.

## Pre-publication history

The pre-publication history for this paper can be accessed here:


